# An Efficient Solid Phase Extraction Method for Purification and Analysis of Compound‐Specific Plant Sugar Stable Hydrogen Isotope Values

**DOI:** 10.1002/rcm.10161

**Published:** 2025-10-28

**Authors:** Selina Hugger, Meisha Holloway‐Phillips, Ansgar Kahmen, Daniel B. Nelson

**Affiliations:** ^1^ Department of Environmental Sciences – Botany University of Basel Basel Switzerland

**Keywords:** compound specific isotope analysis, plant carbohydrates, purification, solid phase extraction, stable hydrogen isotope

## Abstract

**Rationale:**

The stable hydrogen isotope composition (*δ* ^2^H) of plant compounds can serve as environmental or metabolic proxies, but interpretations are hindered by insufficient mechanistic understanding. This can be improved by analyzing the *δ* ^2^H values of metabolic intermediates, such as sucrose, which is the direct substrate of cellulose and a main transport sugar. However, current preparation methods for carbohydrates in general and sucrose in particular are not time‐efficient.

**Methods:**

We evaluated methods that use acetylation of soluble carbohydrate plant extracts to aid in purification as well as isotopic analysis of plant sugars such as sucrose. Extracts were obtained using either hot water or hot 80% ethanol. Acetylated extracts were then purified using an established liquid–liquid separation (LL) method or a new solid phase extraction (SPE) method that we developed. We also evaluated glucose produced from starch after enzymatic digestion. Method performance was evaluated based on quantified yields and the impact on measured *δ* ^2^H values.

**Results:**

Acetylated sucrose and starch‐derived glucose were sufficiently resolved for gas chromatography in all cases. No isotopic biasing was detected for any method. Acetylated sucrose yields differed among methods, with 80% ethanol resulting in approximately threefold higher extraction yield compared to water, and SPE giving smaller but still sufficient yields compared to LL. Sample throughput was doubled with the SPE method compared to the LL method, which allows for larger batch sizes compared to LL.

**Conclusions:**

We developed an efficient method to analyze compound‐specific plant carbohydrate *δ* ^2^H values using gas chromatography‐isotope ratio mass spectrometry (GC‐IRMS). This can be applied in experiments aimed at investigating the processes that shape cellulose *δ* ^2^H values, including deconvoluting the metabolic and hydro‐climatic sources of isotopic variation.

## 1 Introduction

The hydrogen stable isotope composition (*δ* ^2^H) of organic compounds synthesized by plants is first derived from that of the water in plants, including the water taken up by roots and the evaporative processes that occur primarily in leaves. Therefore, stable hydrogen isotopes in plant compounds such as cellulose have been used as a proxy to investigate the hydrological environment as well as plant physiological processes influencing transpiration [1, 2, 3, 4]. In addition to this information, the metabolism of the plant also influences plant organic *δ* ^2^H values, resulting in ^2^H‐offsets between plant water and cellulose *δ* ^2^H values. This is because isotopic fractionation during enzymatic reactions in C metabolism and biosynthetic pathways is not constant but varies with different growth conditions [5, 6, 7, 8, 9, 10, 11, 12, 13, 14, 15, 16]. While these metabolic processes complicate the interpretation of the hydrological signal in cellulose *δ* ^2^H, the metabolic component is of particular interest to understand how plant C metabolism responds to changing environmental conditions and, in turn, influences plant performance [10, 11, 17, 18, 19, 20, 21].

To better understand cellulose *δ* ^2^H variation, resolving *δ* ^2^H values of intermediate compounds along the biosynthetic pathway from photoassimilates to cellulose is essential. This provides an opportunity to deconvolute net biosynthetic isotopic fractionation observed for cellulose into discrete processes occurring during initial sugar synthesis in source leaves and sink‐cell metabolic processes. Sucrose is an attractive intermediate compound for this purpose because it is a common transport sugar and the main substrate for cellulose synthesis [20, 22, 23]. In fact, it was found that two‐thirds of the variation observed in leaf cellulose *δ* ^2^H across species and photosynthetic types is caused by variation in leaf sucrose *δ* ^2^H [17, 24]. Therefore, measurement of sucrose *δ* ^2^H values can help to parameterize a generalized two‐component model describing cellulose *δ* ^2^H as a mixture of “photosynthetic” (source leaf metabolism) and “post‐photosynthetic” (post sugar export) isotope fractionation processes [3, 17]:

$$\begin{array}{c} \delta^{2}H_{\text{cellulose}}=\left(1-f\right)\;\left[\delta^{2}H_{\text{leafwater}}\;\left(1+\varepsilon_{A}\right)+\varepsilon_{A}\right]\\ \;+f\;\left[\delta^{2}H_{\text{xylemwater}}\;\left(1+\varepsilon_{H}\right)+\varepsilon_{H}\right] \end{array}$$

where *f* describes the fraction of carbon‐bound hydrogen in sugars that has undergone isotopic exchange with water during sink cell metabolism and before cellulose synthesis, ε_A_ is the isotope fractionation during photosynthetic reactions, and ε_H_ the net isotope fractionation during post‐photosynthetic reactions. Sucrose *δ* ^2^H values can be used to approximate the photosynthetic contribution to cellulose *δ* ^2^H (i.e., the term [*δ* ^2^H_leafwater_ (1 + ε_A_) + ε_A_]), which has been shown to differ between species [17]. However, despite the interest in investigating sucrose *δ* ^2^H, sucrose extractions are time‐consuming, and these methodological inefficiencies hinder widespread application.

There are two issues that need to be addressed when measuring sucrose *δ* ^2^H values. Firstly, the presence of hydroxyl hydrogen, which can isotopically exchange with surrounding liquid water or water vapor (e.g., during sample processing and analysis) [25], obscures the primary plant hydrological and metabolic information contained in the isotopic values of the non‐exchangeable carbon‐bound hydrogen. Secondly, sucrose needs to be separated from other plant compounds (e.g., other carbohydrates, including other sugars).

One possible solution to deal with exchangeable hydrogen is the equilibration of samples with water vapor of known isotopic composition, known as dual equilibration techniques [26, 27, 28]. However, this requires near‐perfect purification of a single target compound for compound‐specific isotope analysis, and it can also be difficult to achieve an exchange of all hydrogen atoms in the molecule depending on sample type (e.g., crystalline solids, sugars) [29]. Another possibility is to permanently replace the exchangeable hydrogen of the target compound with other molecular groups, which do not contain exchangeable hydrogen.

There are two derivatization methods that have so far been utilized for stable hydrogen isotope analysis of plant sugars: trifluoroacetylation (TFA) [30, 31, 32, 33, 34] and acetylation [17]. Both methods have the advantage that they increase the volatility of the compounds and thereby render them amenable for analysis by GC. Also, in contrast to equilibration techniques, samples analyzed by GC‐IRMS do not need to be perfectly purified, since only volatile substances are transferred to the column, and the different volatile compounds elute at different times. The GC measurement also has the advantage of requiring relatively low sample amounts (~2 μg sucrose on column). The analysis on a GC separates the sugars from each other and thus provides the opportunity to assess the sugar composition of a sample as well as to make compound‐specific stable isotope measurements of single sugars.

While TFA has the advantage that it does not add additional hydrogens to the compound, it requires modification of the pyrolysis reactor for isotope analysis using GC‐IRMS because of the halogens in the samples, and TFA derivatives are also less stable than acetylated compounds [31, 32, 33, 34]. Thus, although acetylation adds a substantial amount of H that must be accounted for and thereby increases the analytical uncertainty of measured *δ* ^2^H values, the practical advantages make this method particularly attractive to plant‐based isotope investigation.

In the acetylation method used by Holloway‐Phillips et al. [17], bulk plant sugars were first extracted using hot water. The whole dried sugar extract was then acetylated using acetic anhydride and pyridine. After drying off the acetylating reagents, the sample was cleaned with a liquid–liquid separation (LL). As this is a time‐consuming process, we aimed to optimize this previously established method for higher throughput. For this, we targeted two critical points. Firstly, we assessed the solvent for the initial extraction of soluble plant sugars. While water is one option for extracting sugars from plant material [17, 35, 36, 37, 38], it has been suggested that 80% ethanol is preferable because—along with sugars—starch can also partially dissolve in water [39]. Another potential advantage of using ethanol is the possibility of measuring starch *δ* ^2^H values from the sample material that is left after sugar extraction, i.e., reducing the carbohydrate extraction requirement from two to one. However, the effect of the choice of extraction solvent on extracted carbohydrate yields and *δ* ^2^H values has so far not been systematically evaluated. In the first step of the current study, we therefore compared the extraction of soluble sugars from leaf material using either water or 80% ethanol.

Secondly, the liquid–liquid separation step performed after acetylation poses a bottleneck because the evaporation process is slow, and liquid–liquid separations are both time‐consuming and error‐prone. To address these points, we developed an alternative purification method to extract the acetylated sugars from the remaining plant sample matrix using reverse phase solid phase extraction (SPE) columns. SPE has been used in the past to purify other organic compounds from various sample matrices [40, 41, 42]. With the potential to separate some of the derivatization agent and solvent from the acetylated sugars, the expected benefits of SPE in contrast to liquid–liquid separation are that it offers easier handling, faster sample throughput, and the possibility for automated sample preparation.

In this work, we therefore tested these two critical points—carbohydrate extraction solvent and purification by SPE columns—and evaluated them by performing parallel extractions and purifications, and then comparing yields and *δ* ^2^H values of sucrose.

## 2 Methods

### 2.1 Chemicals

We used different types of starting material for different purposes during method optimization. Firstly, retention times of individual acetylated sugars during gas chromatography were confirmed by comparing to purchased sugars that we acetylated with 50 μL each of acetic anhydride (Sigma, prod. No. 320102, ReagentPlus, ≥ 99%) and pyridine (Carl Roth, prod. No. 9729.3, ROTIPURAN, ≥ 99.5%) per 100 μg pure sugar (D‐(+)‐Glucose, Sigma, prod. No. G8270, ≥ 99.5%; D‐(−)‐fructose, Sigma, prod. No. F0127, ≥ 99%; D‐(+)‐Galactose, Sigma, prod. No. G0625, ≥ 98%; sucrose, Sigma, prod. No. S1888, ≥ 99.5%), noting that the use of less pure pyridine (Sigma, prod. No. P57506, ReagentPlus, ≥ 99%) can cause chromatography issues (Figure S1). Secondly, purchased stock sugar acetates (Sigma, α‐D(+)‐Glucose pentaacetate, G2354, 99%; Sigma, sucrose octaacetate, W303801‐SAMPLE‐K, ≥ 97%, FG) were used to evaluate the general conditions needed to retain sugar acetates on the SPE sorbent. Thirdly, we used dried and ground plant material to test the conditions needed to purify acetylated plant carbohydrate extracts using SPE.

Once an SPE method was established for plant samples, we tested the use of water versus 80% ethanol as an extraction solvent for the initial hot solvent extraction of sugars from milled plant powder [39]. Finally, we purified acetylated plant carbohydrate extracts from the same plant powder with the established liquid–liquid separation method to compare with our new SPE method.

All polyethylene and polypropylene items were cleaned by sonication first in de‐ionized water and then in ethanol, and all glassware was thermally cleaned in a combustion oven for 4 h at 450°C.

### 2.2 Sugar Extraction and Acetylation

To simulate the solvent system for plant carbohydrate extracts, we treated the stock sugar acetates (200 μg each of sucrose octaacetate and glucose pentaacetate per sample) with 5 mL each of acetic anhydride and pyridine using the established procedure [17]. Importantly, these relatively large reagent volumes are required for plant carbohydrate extracts to ensure that no isotopic biasing is introduced due to only partial completion of the derivatization. The glass vials were sealed under nitrogen gas flow to minimize the amount of air that could impair the reaction. The samples were then mixed by vigorous shaking and placed in an oven at 50°C for 2 h to facilitate acetylation.

To apply the SPE method to plant material, we used broad bean ( *Vicia faba* L.) leaves from plants grown under controlled conditions as testing material. Upon harvest, the leaves were microwaved for 30 s to stop enzymatic activity, oven‐dried at 55°C, and ground in a ball mill (MM400, Retsch GmbH, 42 781 Haan, Germany). Sugars were extracted from 20 mg of fine powder per sample with 1.5 mL ultrapure water at 85°C for 20 min [17]. As a general recommendation, an initial plant dry weight of 20 mg is a good starting point for most sample types in our experience, which can be adjusted if the sugar and starch contents are known. Once the SPE method was established (see below), we tested the use of 80% ethanol as extraction solvent instead of water [39]. Samples were then mixed by shaking, centrifuged (3 min, 15 000 rcf, RT), and the supernatant was taken and filtered with polyethersulfone filters (0.45 μm). The filtered extract was transferred to 20 mL glass vials. The 85°C extraction was repeated one more time, and the resulting 3 mL extract was dried in an oven at 55°C. If starch *δ* ^2^H values are targeted, the starch can be purified from the pellet left after soluble sugar extraction. After digesting the starch with enzymes, the starch‐derived glucose can be acetylated and purified like a soluble sugar sample [43]. Once dry, the bulk plant carbohydrate extracts were acetylated as described above with 5 mL acetic anhydride and pyridine each, at 50°C for 2 h.

**FIGURE 1:**
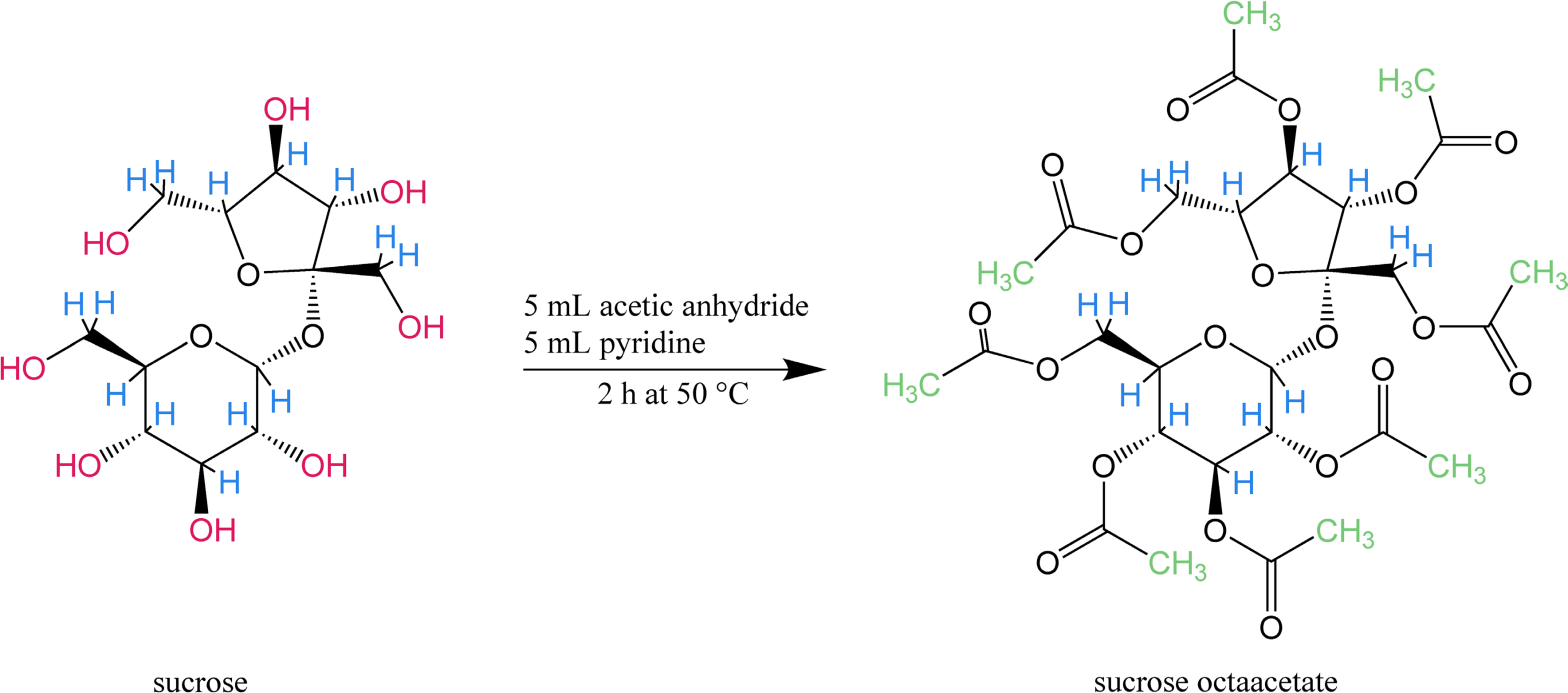
Schematic acetylation reaction of sucrose to sucrose octaacetate. All exchangeable hydrogen (—OH, red) is replaced by acetyl groups containing only non‐exchangeable (carbon‐bound) hydrogen (green). Original carbon‐bound hydrogens are depicted in blue.

During acetylation, each hydroxyl hydrogen is replaced by an acetyl group, each of which contains three carbon‐bound hydrogens (Figure 1). The contribution of this added H to the whole molecule *δ* ^2^H value must be accounted for, so it is necessary to determine the *δ* ^2^H of the acetic anhydride. This can be measured directly as a liquid sample on a thermal conversion elemental analyzer (TCEA) connected to an IRMS [44], or it can be calculated after acetylating two aliquots of the same sugar with two different acetic anhydrides—one being the lab working stock and the other being acetic anhydride with a predetermined *δ* ^2^H value. From these measurements, the *δ* ^2^H value of the unknown acetic anhydride can be calculated [17]. For TCEA‐IRMS measurements, to compensate for small differences between water‐based working standards and acetic anhydride samples, the known *δ* ^2^H value acetic anhydride is also analyzed so that resulting *δ* ^2^H values can be shifted by an appropriate amount (typically 3‰–4‰) after the values are normalized to the Vienna Standard Mean Ocean Water—Standard Light Antarctic Precipitation (VSMOW‐SLAP) scale using the water standards.

### 2.3 Sample Pre‐Treatment for SPE

We aimed to develop a method that would allow acetylated samples to be added to the SPE columns without evaporating the solvents first, a step that is needed for the liquid–liquid separation and usually takes several hours. To achieve this, we diluted both the stock sugar acetates and the plant carbohydrate extracts directly after acetylation with 50–65 mL of pre‐cooled ultrapure water. The water was pre‐cooled to prevent excessive heating due to the exothermic reaction with the acetic anhydride and acetic acid (reaction byproduct) in the sample vial. This dilution makes the solvent solution more polar and thereby increases the retention of the sugar acetates on the SPE sorbent. In an initial testing phase, limited tests (with two to three replicates) were performed to evaluate the necessary dilution volume and the effect of acidifying the sample solution on sugar acetate yields. These limited tests were done on three different SPE sorbents (each with 0.5 g of sorbent per column), two relatively common end‐capped modified silica gels of different chain lengths (ISOLUTE C8ec, Chromabond C18ec), and one hydrophobic spherical polystyrene‐divinylbenzene copolymer (Chromabond HR‐X). Based on this comparison (Figures S2, S3), we focused further tests on the C18ec sorbent. We increased the amount of sorbent to 2 g to ensure that there were enough active sites for sugar acetates in bulk plant carbohydrate extracts.

### 2.4 SPE

SPE columns were set up on a vacuum block and conditioned with 12 mL methanol and equilibrated with 12 mL ultrapure water. The diluted samples were then added onto the columns using 70 mL reservoirs, and the eluted liquid was collected as waste. After the sample liquid was eluted to the top of the stationary phase, the acetylated sugars were eluted from the sorbent using 20 mL acetone and collected in 20 mL glass vials. A second fraction of 20 mL acetone was collected (which should also be done for new sample types with unknown sugar content) to ensure complete elution of the compounds from the sorbent. The fractions were then dried under nitrogen flow and transferred to 1.5 mL autosampler vials using dichloromethane, dried again, and re‐diluted in 400 μL acetone for measurement on a gas chromatography‐flame ionization detector (GC‐FID) for quantification of sugar acetates.

We optimized the elution of sugar acetates by testing different elution solvents (acetone and methanol) and the use of a vacuum pump for faster elution. When using a vacuum pump, the flow speed was adjusted to 1–2 drops per second with a mild vacuum. Again, the final fractions were dried, transferred to 1.5 mL autosampler vials, and re‐diluted in acetone for measurement on a GC‐FID for quantification and GC‐IRMS for hydrogen isotopic analysis.

Additional validation of the final SPE method was done on leaves of broad bean ( *V. faba* L.), radish ( *Raphanus sativus* L.), and sunflower ( *Helianthus annuus* L.), leaves and roots of tobacco ( *Nicotiana sylvestris* Speg. & Comes), as well as leaves and wood of several tree species ( *Betula pendula* Roth, *Carpinus betulus* L., *Fagus sylvatica* L.).

### 2.5 Liquid–Liquid Separation

For the liquid–liquid separation, acetylating reagents were removed by evaporation directly after acetylation. Additional sample cleanup was still required after this step, however, to remove aqueous‐soluble material that interfered with sample solubility in organic solvents. This was done in three liquid–liquid separation rounds using 5 mL each of heptane‐cleaned ultrapure water and 2:1 heptane/dichloromethane, with acetylated sugars recovered in the organic phase and water‐soluble co‐extracts separated into the aqueous phase [17]. The organic phase was collected, dried under nitrogen gas flow, transferred to 1.5 mL autosampler vials, and re‐diluted in acetone for measurement on a GC‐FID for quantification and GC‐IRMS for hydrogen isotopic analysis.

### 2.6 Quantification of Sugar Acetates

All acetylated sugars were analyzed by GC‐FID (Trace 1310; Thermo Fisher Scientific) to assess the yields of the different methods. Yields were calculated as the ratio of obtained sugar acetate assessed by GC‐FID relative to the initial amount of either stock sugar acetates (yields then expressed as a percentage) or plant powder (expressed as μg sugar acetate per mg dry weight). Peaks were identified by comparison of retention times to purchased sugar acetates as reference standards. Quantifications were performed using 6‐point calibration curves and checked for accuracy using the same reference standards; the calibration curves had *R* ^*2*^‐values of 0.998 and 0.977 for sucrose octaacetate and glucose pentaacetate, respectively. The GC‐FID was equipped with a programmable temperature vaporization inlet operated in splitless mode, and an Rtx‐5 ms column (Restek; 30 m × 0.25 mm × 0.25 μm). The GC oven program was held at 40°C for 2 min, then ramped to 140°C at 15°C/min, then ramped to 325°C at 10°C/min and held for 10 min. Helium carrier was used at a constant flow rate of 1.2 mL/min.

### 2.7 Assessment of Sample Composition

A subset of samples was also analyzed on a gas chromatography–mass spectrometer (GC–MS) (Trace GC Ultra with DSQII, both Thermo Fisher Scientific, Waltham, MA, USA) to assess the purity of acetylated sugars and to identify any additional compounds that were present in the samples. The GC–MS was equipped with a split/splitless inlet operated in splitless mode at 300°C. The initial oven temperature was 50°C for 2 min, then ramping up to 300°C at 10°C/min. After holding this temperature for 17 min, the temperature was ramped up to 320°C at 10°C/min and was held for 10 min. The column effluent was transferred to the MS via a transfer line at 260°C. Individual compounds were identified using the digital database of the National Institute of Standards and Technology (NIST Mass Spectral Search 2.0, MD, USA) and the Xcalibur GC software (v2.2 SP1.48, Thermo Fisher Scientific Inc., 1998–2009, Waltham, MA, USA).

### 2.8 Measurement of Sugar *δ* ^2^H Values

Approximately 1.7–2 μg of sucrose (or 0.13 μg of H) was injected onto the GC‐IRMS column, using a split/splitless inlet operated in splitless mode on a Trace GC Ultra equipped with a Restek Rtx‐5MS column (30 m × 0.25 mm × 0.25 μm). Like in the GC‐FID method, the initial oven temperature was 40°C for 2 min with helium carrier gas set to a constant flow rate of 1.2 mL/min, then ramping to 140°C at 15°C/min. The temperature was then increased to 320°C at 8°C/min and held for 5 min. The column effluent was introduced to the pyrolysis reactor in a GC‐IsoLink where it was converted to H_2_ at 1420°C, which was then measured on a Thermo Delta V Plus IRMS. At the start of each sequence, the H_3_^+^ factor was determined. It was stable over time during the analyses of the present study and below the manufacturer‐defined threshold. To normalize the measured *δ* ^2^H values to the VSMOW‐SLAP scale, we used a mixture of *n*‐alkanes as well as a C20:0 fatty acid methyl ester with known hydrogen isotopic compositions (Mix A7 and USGS 71, purchased from Arndt Schimmelmann, Indiana University). The long‐term analytical precision was tracked by repeated measurements of a sucrose octaacetate quality control. This sucrose octaacetate quality control was also measured as a bulk sample using a TCEA connected to a separate Delta V Plus IRMS. The TCEA was equipped with a chromium reactor operated at 1030°C [45]. These bulk analyses yielded a value of −105.8‰ ± 0.1‰ (mean ± standard deviation, with *n* = 3), which was in close agreement with the GC‐IRMS‐based value of −109.1‰ ± 3.8‰ (*n* = 212), with the mean difference between those two methods (3.3‰) being smaller than the typical measurement error for hydrogen.

### 2.9 Terminology

The measured stable isotope values were corrected for the added H from the acetic anhydride to get the isotopic composition of the non‐exchangeable H from only the original sugar as follows:

$$F_{\text{sample}}=\frac{R_{\text{sample}}}{R_{\text{sample}}+1}$$

With R being the isotopic ratio (^2^H/^1^H). And then:

$$F_{\text{sugar}}=\frac{N_{M}F_{M}-N_{\mathrm{AA}}F_{\mathrm{AA}}}{N_{\text{sugar}}}$$

With N being the number of hydrogen atoms (with N_M_ = N_sugar_ + N_AA_), subscript M referring to measured values, and AA to acetic anhydride.

Stable isotopic compositions are then given as delta (*δ*) values in ‰ notation, with

$$\delta_{\text{sample}}=\frac{R_{\text{sample}}}{R_{\mathrm{std}}}-1$$

where R_std_ is the stable isotope ratio of VSMOW [46].

### 2.10 Statistics

Analysis of data was done using R version 4.3.3 [47] with RStudio 2023.12.1 + 402 [48]. Packages used were ggplot2 [49], readxl [50], plyr [51], dplyr [52], reshape2 [53], tidyr [54], car [55], forcats [56], gridExtra [57], and ggpubr [58]. Two‐way ANOVAs were done to assess differences in sucrose yield and *δ* ^2^H values between method parameters (extraction solvent, purification method; *n* = 3 per group) after ensuring that the residuals were normally distributed (using a Shapiro–Wilk normality test) and the variance homogeneous (using Levene's Test for Homogeneity of Variance). The interaction term between extraction solvent and purification method was only kept if significant. Figure 1 was made using ChemDraw 23.1.1.

## 3 Results

### 3.1 Chromatography

**FIGURE 2:**
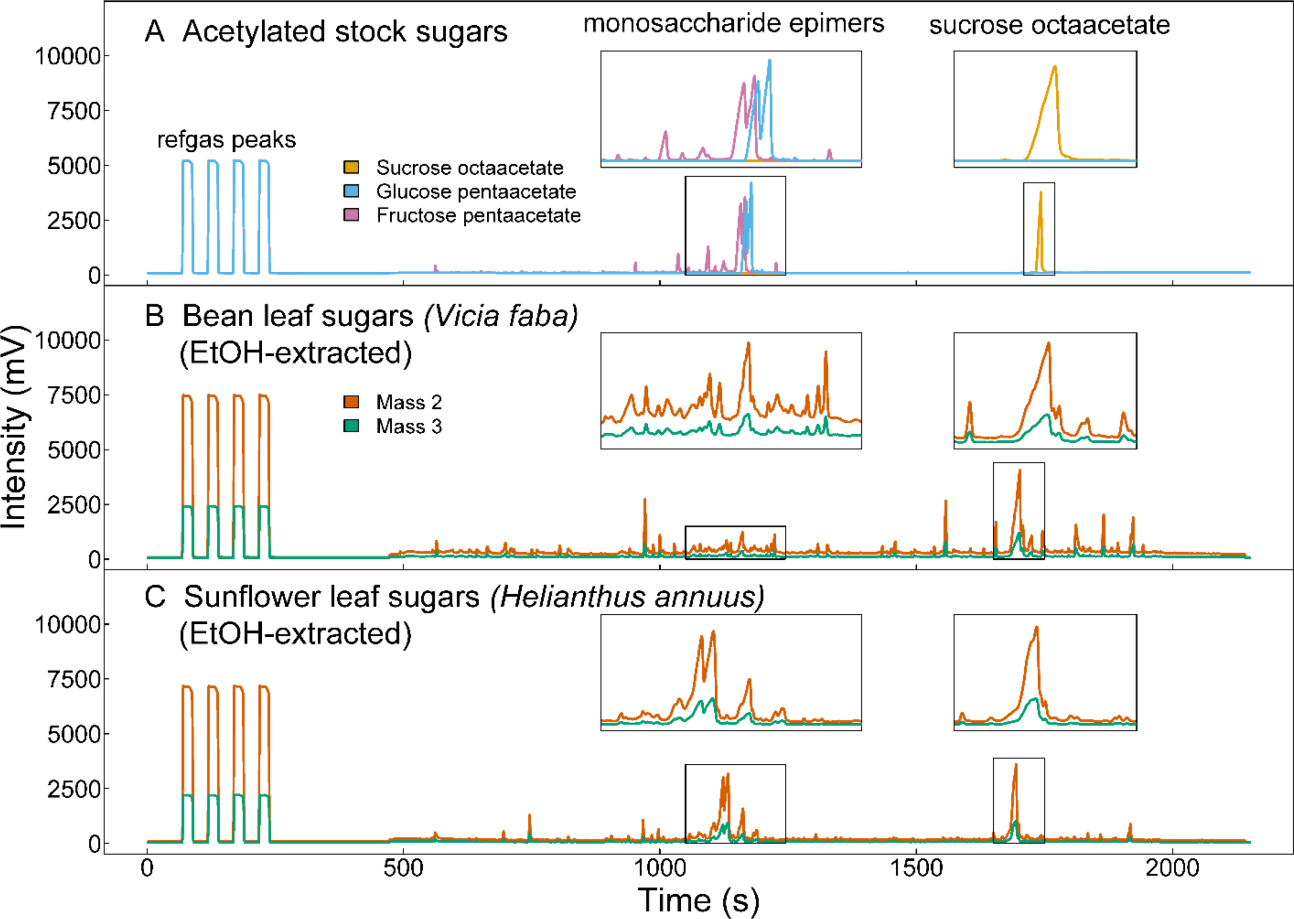
Typical GC‐IRMS chromatograms of acetylated sugars. Acetylated stock sugars (A) and plant leaf non‐structural carbohydrates from bean ( *Vicia faba* , B) and sunflower ( *Helianthus annuus* , C) which were extracted with a hot solvent extraction using 80% ethanol, acetylated, and purified using reverse‐phase C18ec SPE columns. Reference gas peaks, the region of acetylated monosaccharide epimers, and sucrose octaacetate are indicated and the latter are highlighted with insets.

**FIGURE 3:**
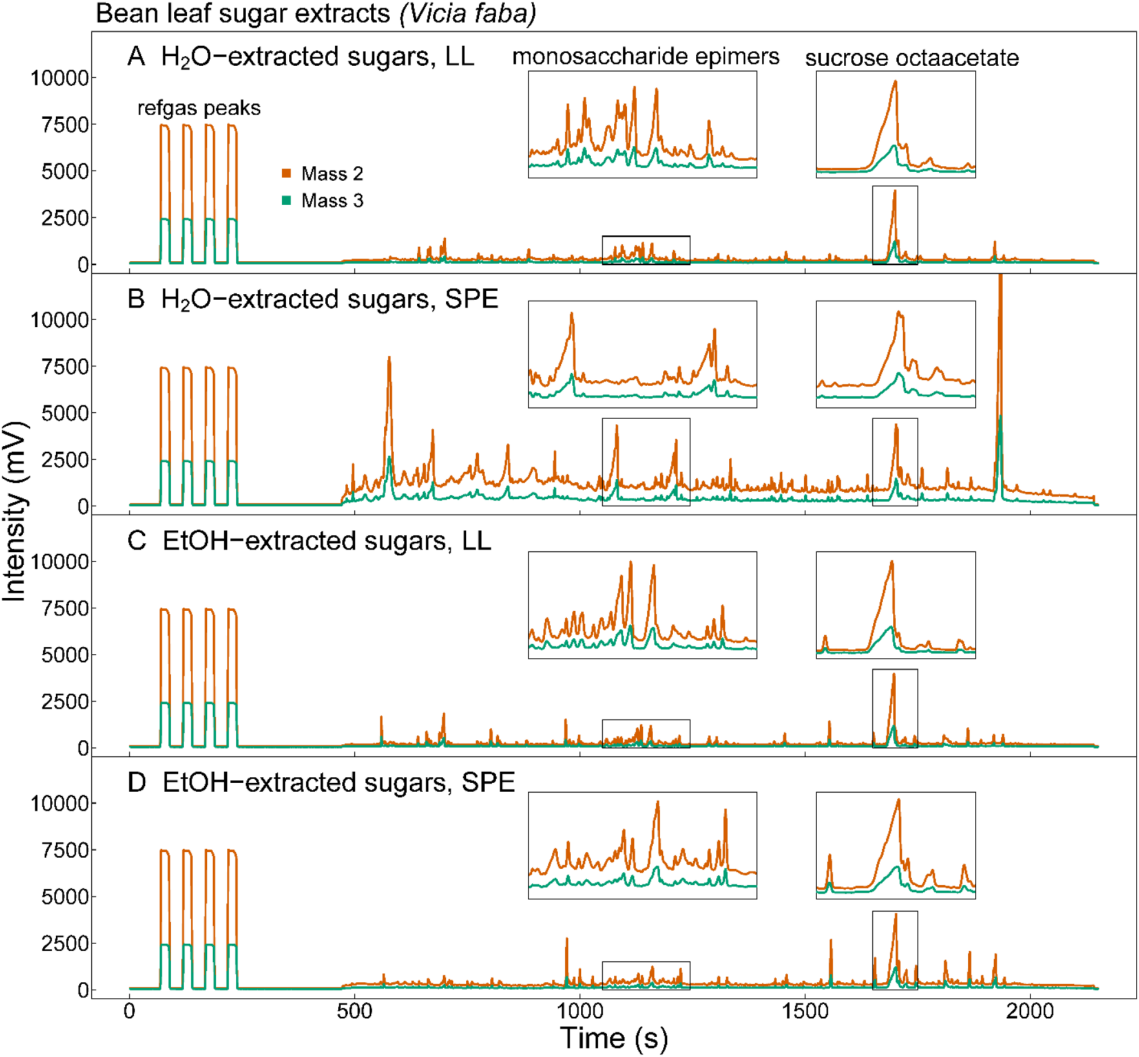
Example chromatograms of acetylated plant carbohydrate extracts produced with the tested methods. Shown are chromatograms of soluble sugar extracts from bean leaves ( *Vicia faba* ). The solvent used for the hot solvent extraction of soluble sugars (nanopure water (H_2_O) (A, B) or 80% ethanol (EtOH) (C, D)), and the purification method that followed acetylation (liquid–liquid separation (LL) (A, C) or solid phase extraction (SPE) (B, D)) are indicated in the header of each chromatogram. Reference gas peaks, the region of acetylated monosaccharide epimers, and sucrose octaacetate are indicated and the latter are highlighted with insets.

As there are different epimers of each sugar present in both purchased pure sugars as well as plant samples, acetylation produces multiple acetylated epimeric forms reflective of the different epimers. The different epimers of a given sugar acetate elute at slightly different retention times during gas chromatography. In the case of the monosaccharides, this results in multiple peaks (Figure 2A) [59]. The integration of the monosaccharide peaks is further complicated by the fact that different monosaccharides (e.g., acetylated glucose and fructose) elute at similar retention times, which can result in co‐eluting peaks (Figure 2A). In contrast, all sucrose octaacetate epimers elute in a single chromatographic peak that can be clearly identified and integrated (Figure 2). In addition to the monosaccharide and sucrose peaks, other peaks were observed, especially in plant samples extracted with 80% ethanol. Some of these peaks are from the solvent system or chromatography hardware (see Figure S4). Further, a subset of samples was analyzed by GC–MS to identify those substances, which included other acetylated carbohydrates, alcohols, and pigments that naturally occur in plants, as well as degradation products from acetic anhydride and siloxane contaminants (usually from the septa of vial caps) (Figure S5). The baseline separation in plant samples was as good as in acetylated pure sugars. However, we found that the yields of carbohydrate samples extracted with water and then purified with SPE were low (~0.7 μg sucrose octaacetate per mg initial dry weight). Therefore, the separation between peaks and baseline was less clear and complicated peak integration (Figure 3B).

### 3.2 Choice of Extraction Solvent

**FIGURE 4:**
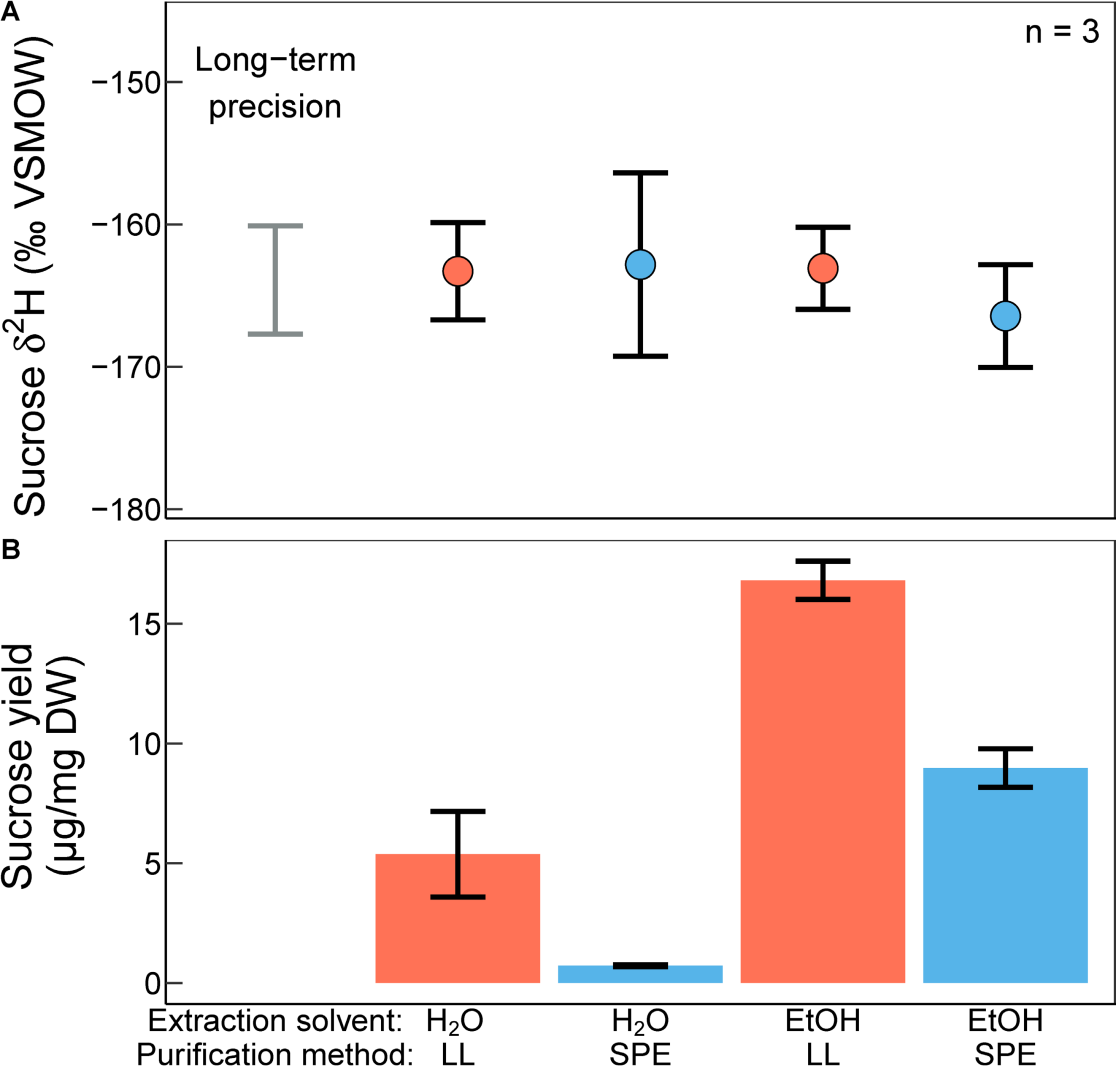
Method comparison. Stable hydrogen isotope values (*δ* ^2^H, A) and yields (μg/mg dry weight (DW), B) of sucrose octaacetate from bean leaves ( *Vicia faba* ) across different methods of extraction solvent (water (H_2_O) or 80% ethanol (EtOH)) and purification (solid phase extraction (SPE) or liquid–liquid separation (LL)). Displayed are mean and standard deviation of *n* = 3 replicates. The grey bar on the left in panel A indicates the long‐term precision for GC‐IRMS measurements (3.8‰), using pure sucrose octaacetate as QC standard with *n* = 212.

Soluble sugars were extracted from dry plant powder using either ultrapure water or 80% ethanol. The choice of extraction solvent had no effect on the sucrose *δ* ^2^H value (Figure 4A), which was confirmed with a two‐way ANOVA (*p* = 0.501, see Table S1). However, using 80% ethanol increased the sucrose yields by approximately threefold for samples purified by liquid–liquid separation and twelvefold for SPE‐purified samples (*p* < 0.001, with *p* = 0.029 for the interaction term between extraction solvent and purification method, Figure 4B, Tables S1 and S2).

### 3.3 Sample Clean‐Up After Acetylation

After acetylation, the derivatized sugars were further purified using either liquid–liquid separation or solid‐phase extraction with reverse‐phase C18ec columns. Both purification methods resulted in statistically identical sucrose *δ* ^2^H values (*p* = 0.568), confirming that the sample clean‐up does not cause hydrogen isotope fractionation in the sugar acetate. The liquid–liquid separation resulted in higher yields than the solid‐phase extraction (approximately twofold for ethanol‐extracted samples and sevenfold for water‐extracted samples with *p* < 0.001, and *p* = 0.029 for the interaction term between extraction solvent and purification method, Figure 4B, Tables S1 and S2).

**FIGURE 5:**
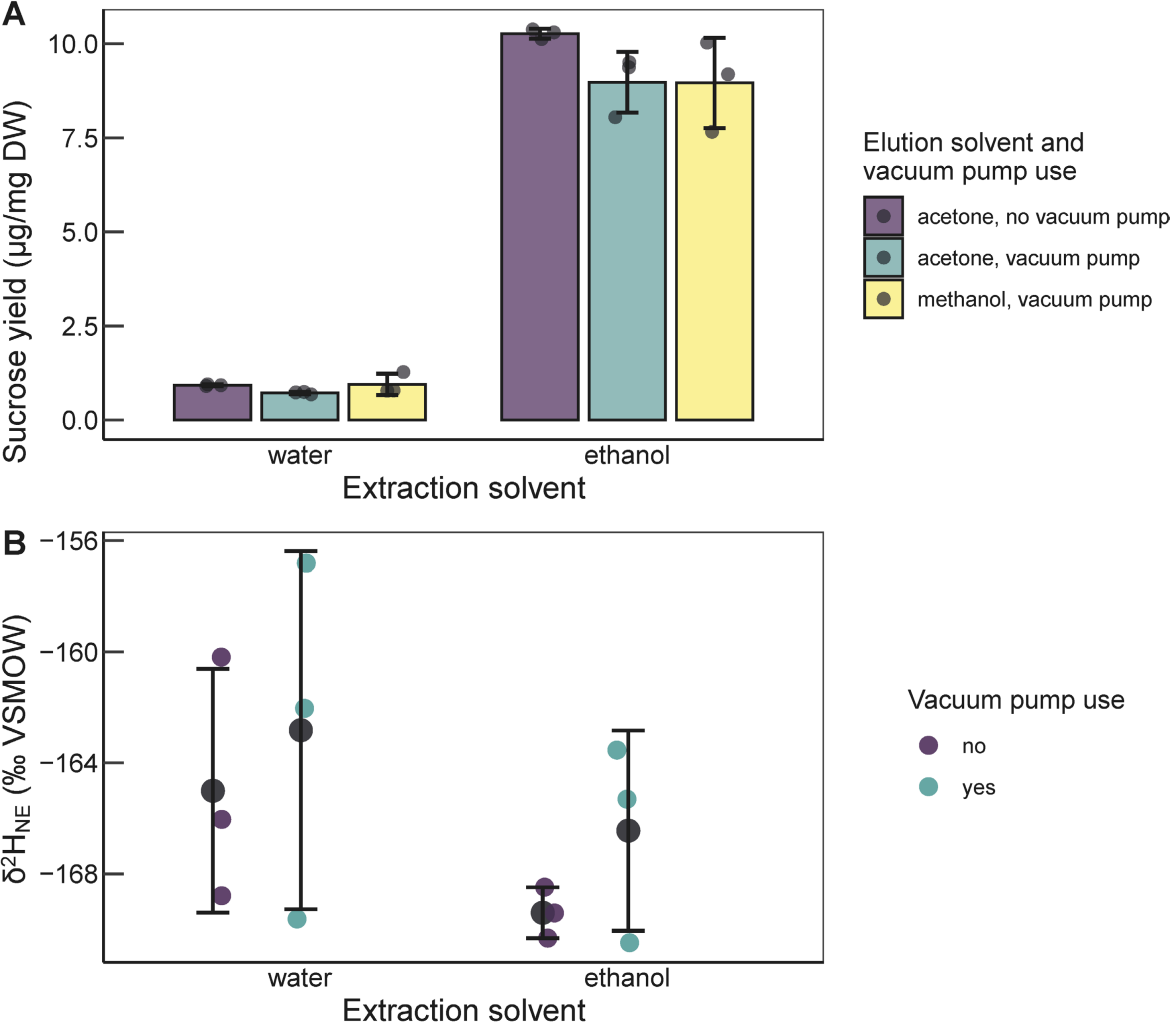
Effect of extraction solvent, elution solvent, and vacuum pump use on yields and *δ* ^2^H values of sucrose octaacetate. Two extraction solvents (nanopure water, 80% ethanol) for the initial hot solvent extraction of soluble carbohydrates from bean leaf powder were tested. Additionally, two elution solvents (acetone, methanol) were tested for the elution of sugar acetates from C18ec SPE sorbent. Also, the effect of the use of a vacuum pump during the SPE procedure was tested. Yields (A) were calculated as the amount of sucrose octaacetate measured by GC‐FID relative to the initial plant dry weight. Sucrose octaacetate *δ* ^2^H values (B) were measured for the sample set with acetone as elution solvent. Displayed are mean and standard deviation of *n* = 3 replicates. The data from the “acetone, vacuum pump” sample set (green) is the same as the one depicted for SPE in Figure 4, just plotted differently.

In the limited tests done to further optimize the SPE method, we found that adjustment of the diluted sample to pH 2 did not improve the yield of pure sugar acetates (Figure S2). Further, a dilution volume of 50 mL to add to the derivatized sample was found to be necessary to achieve optimal yields of sugar acetates from the SPE sorbent (Figure S3). We also found that there was no difference in yields between using acetone or methanol as elution solvent, so we advise acetone due to the faster evaporation of solvent afterwards (Figure 5A). The use of a vacuum pump had no effect on sucrose yields or sucrose *δ* ^2^H values (Figure 5), although this would likely cease to be the case if a very strong vacuum were to be applied.

The final SPE method (Notes S1) was tested on soluble sugar and starch extracts from other plant species (Figure S6) and proved to work well, although it is noteworthy that samples with a high ratio of monosaccharides to sucrose can complicate chromatography (Figure S7). For starch extracts, the glucose pentaacetate peak can be interpreted clearly because no other (co‐eluting) monosaccharides are present in starch‐derived glucose samples.

## 4 Discussion

In this study, we developed a new solid‐phase extraction method for the purification and analysis of compound‐specific plant sugar stable hydrogen isotope values by GC‐IRMS. Further, we compared the use of ultrapure water versus 80% ethanol for the extraction of soluble sugars from bulk plant material. Plant sugar extracts were then acetylated and subsequently purified with either an established liquid–liquid separation method or our new reverse‐phase solid‐phase extraction method. All tested methods resulted in interpretable chromatograms, and there were no significant differences in *δ* ^2^H values among any methods detected with a two‐way ANOVA (Table S1). In contrast, the yields, assessed by the amount of sucrose octaacetate measured on the GC‐FID relative to initial sample dry mass, were different among the methods.

Both water and ethanol are common solvents used for the initial extraction of soluble sugars from plant material [17, 35, 36, 37, 38, 39, 60, 61, 62, 63]. With 80% ethanol as the solvent for sugar extraction, the sucrose yield was increased by at least threefold compared to using water (Figure 4). This observation might be explained by ethanol dissolving cellular structures more effectively than water, thereby releasing more sugars along with other compounds. This is visible in the chromatograms from ethanol extracts, which showed generally more extraneous but non‐coeluting peaks than water‐extracted samples (Figure 3). Using GC–MS, these additional peaks were identified to be mainly plant‐derived compounds and siloxanes from the workup of the samples (Figure S5). Apart from the better yields of sugars, using 80% ethanol for extraction has the additional advantage that one can extract starch from the same initial sample material [39].

Instead of first purifying a compound of interest from a sample matrix before derivatization and measurement—as has been done in the past, e.g., purifying sugars using ion exchange columns [8, 35, 64, 65, 66]—another option is to use the change in chemical properties of the compounds from derivatization to aid in purification [67, 68, 69]. A disadvantage of this approach is the relatively high amount of derivatization reagents needed for bulk extracts compared to more purified samples, but it can still save time and costs. We therefore aimed to optimize a method to purify acetylated plant sugars. In our case, the increase in hydrophobicity from sugars to sugar acetates enabled separation from co‐extracted water‐soluble compounds by liquid–liquid separation or reverse‐phase solid‐phase extraction.

Compared to the established liquid–liquid separation method, our new SPE method showed lower but still adequate sucrose yields if ethanol is used to extract soluble sugars, and both methods yielded statistically identical *δ* ^2^H values (Figure 4, Tables S1, S2). The combination of water‐extracted sugars purified with SPE resulted in particularly low yields and more difficult peak integration due to a comparatively elevated baseline, and is therefore not recommended, even though the *δ* ^2^H values were similar as with the other methods. We speculate that this observation might be caused by the co‐extracted compounds in water extracts causing larger matrix effects than those in ethanol extracts. Additionally, those matrix effects seem to affect SPE more than LL, explaining the especially low sucrose octaacetate yields from water extracts purified with SPE.

The new SPE method facilitates faster sample handling and throughput. In our experience, sample throughput was doubled with the SPE method compared to the liquid–liquid separation (24 versus 12 samples processed in 2 days, respectively), although we are aware that different laboratory equipment (especially evaporation systems with different numbers of sample slots) can change the throughput of methods like the ones described here. Moreover, the throughput of the SPE method could potentially be further improved with automated systems for the SPE itself and for subsequent drying and transferring steps of collected fractions.

The SPE method was further validated on other plant materials and proved to work well for soluble sugar and starch extracts of leaf, wood, and root material from various plant species (*V. faba, R. sativus*, *H. annuus, N. sylvestris, B. pendula, C. betulus, F. sylvatica*) (Figure S6). However, samples with an extremely high ratio of monosaccharides to sucrose pose a difficulty for achieving good chromatography (Figure S7). Even though the compounds are well resolved, the total sample required to achieve adequate peak sizes for sucrose overwhelms the inlet and leads to delayed transfer of all compounds onto the GC column. For such samples, it might be an option to first separate sucrose from monosaccharides, for example, by using HPLC [62, 63, 65, 70, 71, 72].

To get the *δ* ^2^H values of the non‐exchangeable hydrogen from only the original sugar, the *δ* ^2^H value of the hydrogen added during acetylation must be accounted for. Since this is approximately two‐thirds of total hydrogen for sucrose octaacetate and starch‐derived glucose pentaacetate, this adds to the analytical error of the final *δ* ^2^H values. An alternative derivatization method that circumvents this issue is trifluoroacetylation [30, 33, 34]. In this method, the exchangeable hydrogen is replaced with trifluoroacetyl groups, which do not add any hydrogen atoms to the molecule. However, the TFA method requires special instrument modification because of the halogens contained in the samples, and the products are also not as stable as acetylated compounds, meaning derivatization must be performed directly prior to isotope analysis [31, 32].

Based on our findings, we recommend using 80% ethanol as an extraction solvent and SPE for purification after acetylation (see Notes S1 for the full procedure). If time or labor resources allow for it, and especially for samples with a particularly low soluble sugar content (< 1%), liquid–liquid separation is preferable because the yields are higher than with SPE. This strategy should ensure sufficient yields while facilitating a fast sample throughput.

## 5 Conclusion

With this work, we present an improved and efficient method to analyze compound‐specific plant carbohydrate *δ* ^2^H values on GC‐IRMS, with sucrose octaacetate and starch‐derived glucose pentaacetate being well resolved under normal GC measurement conditions and on commonly available GC columns. The ability to measure a multitude of compounds is beneficial for various research fields, such as plant and aquatic ecology, biogeochemistry, paleoclimatology, and food forensics. Also, this will help in plant physiological experiments aimed at disentangling metabolic from hydrological influences on cellulose *δ* ^2^H values. Such knowledge will advance hydrological studies as well as enhance the understanding of metabolic processes occurring in plants in response to environmental changes. Hence, our method will find applications in experiments investigating plant responses to changing environmental conditions, such as varying water supply, temperature, nutrient supply, and atmospheric CO_2_ concentration.

## Author Contributions

**Selina Hugger:** investigation, methodology, writing – review and editing, writing – original draft, visualization. **Meisha Holloway‐Phillips:** supervision, writing – review and editing. **Ansgar Kahmen:** funding acquisition, writing – review and editing, supervision. **Daniel B. Nelson:** supervision, writing – review and editing, methodology, conceptualization, project administration.

## Supporting information


**Table S1:** Results from two‐way ANOVAs. Differences in sucrose yields and *δ* ^2^H values between method parameters (extraction solvent, purification method; *n* = 3 per group) were tested after ensuring that the residuals were normally distributed (using a Shapiro–Wilk normality test) and the variance homogenous (using Levene's Test for Homogeneity of Variance). Note that the interaction term of the sucrose *δ* ^2^H model was removed as it was not significant.
**Table S2:** Method comparison. Yield and stable hydrogen isotopic composition of sucrose octaacetate from bean leaves ( *Vicia faba* ) across different methods of extraction solvent (water or 80% ethanol) and purification (solid phase extraction or liquid–liquid separation). The yield was assessed as the amount of sucrose octaacetate obtained after purification (measured on a GC‐FID) per initial dry weight. The hydrogen isotopic composition was then measured on a GC‐IRMS and is stated as *δ* ^2^H with associated standard deviation (SD) obtained from repeated measurements (n).
**Figure S1:** Effect of pyridine purity on GC‐IRMS chromatography and *δ* ^2^H values. Whole *V. faba* leaf carbohydrate extracts were acetylated with acetic anhydride and pyridine. For the pyridine, either a high purity pyridine (≥ 99.5%, panel A and yellow points in panel C) or a low purity pyridine (≥ 99.0%, panel B and purple points in panel C) was used. Note the hump in the chromatography (B) at the end of the sequence when low purity pyridine is used, and the difference in the *δ* ^2^H values of sucrose octaacetate (C).
**Figure S2:** Effect of SPE sorbent and pH on sucrose and glucose recoveries. Three SPE sorbents (HR‐X, C8ec, C18ec) were tested with stock sucrose octaacetate (A) and glucose pentaacetate (B) (200 μg each per sample). To simulate the solvent system, 5 mL each of acetic anhydride and pyridine were added, and samples were heated for 2 h at 50°C. Then, samples were diluted and either acidified to pH 2 using HCl (purple bars) or not (pH 5, yellow bars). Recoveries were calculated as the percentage of the compound measured by GC‐FID relative to its initial amount. Shown are the mean and standard deviation of *n* = 2–3 replicates.
**Figure S3:** Effect of SPE sorbent and dilution volume on sucrose yields. Three SPE sorbents (HR‐X, C8ec, C18ec) were tested on acetylated carbohydrate extracts from bean leaf powder (extracted with a hot water extraction). Additionally, samples were diluted after derivatization with either 25 or 50 mL of nanopure water (blue or yellow bars, respectively). Yields were calculated as the amount of sucrose octaacetate measured by GC‐FID relative to the initial plant dry weight. Shown are the mean and standard deviation of *n* = 2–3 replicates.
**Figure S4:** GC‐FID chromatogram of a solvent blank. Acetic anhydride and pyridine (5 mL each) were heated for 2 h at 50°C, diluted with 50 mL ultrapure water, applied to SPE columns, and eluted with 20 mL acetone after the solution was eluted to the top of the stationary phase. A chromatogram of pure sugar acetates is added in grey as reference.
**Figure S5:** Assessment of sample composition. GC–MS chromatogram of a carbohydrate extract from bean leaf material ( *V. faba* ) extracted with 80% ethanol, and after acetylation subsequently SPE‐purified (A). The main peaks are numbered and the region of monosaccharides and sucrose highlighted with grey boxes and enlarged in panels B and C. The peaks were identified as following substances: 1) mannitol‐acetate, 2) siloxane, 3) 3,7,11,15‐Tetramethyl‐2‐hexadecen‐1‐ol, 4) methyl‐tetraacetyl‐α‐D‐mannopyranoside, 5 + 7) D‐glucose‐pentaacetate, 6) D‐Galactofuranose‐pentaacetate, 8) myo‐inositol‐hexaacetate, 9) tetracosanol, 10) cellobiose‐octaacetate, 11 + 13) sucrose‐octaacetate, 12) acetylated glucopyranoside.
**Figure S6:** Example chromatograms of acetylated plant carbohydrate extracts purified with the new SPE method. Shown are chromatograms of soluble sugar extracts and starch extracts. These were produced from leaves of radish ( *Raphanus sativus* ) and sunflower ( *elianthus annuus* ), leaves and roots of tobacco ( *Nicotiana sylvestris* ), as well as leaves and wood of several tree species (*Betula pendula, Carpinus betulus, Fagus sylvatica*). Species, plant organ, and sample type (“sucrose” for soluble sugar extracts, or “starch” for starch extracts) are indicated in the header of each chromatogram.
**Figure S7:** Example chromatograms of acetylated plant carbohydrate extracts with high monosaccharide contents. Shown are chromatograms of soluble sugar extracts from wild‐type and *pgm*‐mutant *N. sylvestris* leaves which contained a high ratio of monosaccharides to sucrose, complicating chromatography.

## Data Availability

The data that support the findings of this study are available from the corresponding author upon reasonable request.
