# Supplementary material for: An Efficient Solid Phase Extraction Method for Purification and Analysis of Compound‐Specific Plant Sugar Stable Hydrogen Isotope Values
Source: Rapid Commun Mass Spectrom. 2025 Oct 28;40(2):e10161. doi: 10.1002/rcm.10161 (PMC12560618; doi:10.1002/rcm.10161)
Supplement: Supplementary file 1 — Table S1: Results from two‐way ANOVAs. Differences in sucrose yields and δ 2H values between method parameters (extraction solvent, purification method; n = 3 per group) were tested after ensuring that the residuals were normally distributed (using a Shapiro–Wilk normality test) and the variance homogenous (using Levene's Test for Homogeneity of Variance). Note that the interaction term of the sucrose δ 2H model was removed as it was not significant. Table S2: Method comparison. Yield and stable hydrogen isotopic composition of sucrose octaacetate from bean leaves ( Vicia faba ) across different methods of extraction solvent (water or 80% ethanol) and purification (solid phase extraction or liquid–liquid separation). The yield was assessed as the amount of sucrose octaacetate obtained after purification (measured on a GC‐FID) per initial dry weight. The hydrogen isotopic composition was then measured on a GC‐IRMS and is stated as δ 2H with associated standard deviation (SD) obtained from repeated measurements (n). Figure S1: Effect of pyridine purity on GC‐IRMS chromatography and δ 2H values. Whole V. faba leaf carbohydrate extracts were acetylated with acetic anhydride and pyridine. For the pyridine, either a high purity pyridine (≥ 99.5%, panel A and yellow points in panel C) or a low purity pyridine (≥ 99.0%, panel B and purple points in panel C) was used. Note the hump in the chromatography (B) at the end of the sequence when low purity pyridine is used, and the difference in the δ 2H values of sucrose octaacetate (C). Figure S2: Effect of SPE sorbent and pH on sucrose and glucose recoveries. Three SPE sorbents (HR‐X, C8ec, C18ec) were tested with stock sucrose octaacetate (A) and glucose pentaacetate (B) (200 μg each per sample). To simulate the solvent system, 5 mL each of acetic anhydride and pyridine were added, and samples were heated for 2 h at 50°C. Then, samples were diluted and either acidified to pH 2 using HCl (purple bars) or not (pH 5, yellow bars). [file RCM-40-e10161-s001.pdf]

Supporting Information for:

An efficient solid phase extraction method for purification and analysis of compound-specific plant sugar stable hydrogen isotope values

Selina Hugger[1], Meisha Holloway-Phillips[1], Ansgar Kahmen[1], Daniel B. Nelson[1]

[1]University of Basel, Department of Environmental Sciences – Botany, Schönbeinstrasse 6, 4056 Basel, Switzerland

Corresponding author: Selina Hugger, se.hugger@unibas.ch

Notes S1:

We suggest the following method for the extraction, acetylation, and purification of plant soluble carbohydrates:

- For most sample types, 20 mg initial dry weight of plant powder is a good starting point, but this can be adjusted if sugar and starch contents are known.
- Plant powder is extracted with 80%-ethanol, two rounds of 1.5 mL for 20 min at 85 °C, and then the extracts are dried.
- Acetylate with 5 mL each of acetic anhydride and pyridine, cap under N_2 flow and seal well, mix, then heat for 2 h at 50 °C. The δ^2H value of the acetic anhydride must be known.
- Add 50 mL cold, ultrapure water. Vortex.
- Condition and equilibrate a 2 g C18ec RP-SPE column with 12 mL methanol and then 12 mL ultrapure water.
- Apply the 60 mL of sample to the column using a large volume reservoir. Use a vacuum pump at 1-2 drops per second to pull the liquid through. Collect as waste.
- Elute the sugar acetates using 20 mL of acetone, then evaporate to dryness.
- Transfer to 1.5 mL autosampler vial using an appropriate solvent, then evaporate to dryness. Re-dilute in a mid-polarity solvent like acetone for GC measurement.

Tables

Table S1: **Results from two-way ANOVAs.** Differences in sucrose yields and δ^2H values between method parameters (extraction solvent, purification method; n=3 per group) were tested after ensuring that the residuals were normally distributed (using a Shapiro-Wilk normality test) and the variance homogenous (using Levene's Test for Homogeneity of Variance). Note that the interaction term of the sucrose δ^2H model was removed as it was not significant.

	df	Sum Sq	Mean Sq	F-value	p
**Formula: d2H.NE ~ extraction.solvent+purification.method**					
extraction.solvent	1	8.75	8.76	0.492	0.501
purification.method	1	6.24	6.24	0.351	0.568
Residuals	9	160.08	17.79		
**Formula: Suc.yield ~ extraction.solvent*purification.method**					
extraction.solvent	1	292.36	292.36	262.84	<.000 ***
purification.method	1	117.41	117.41	105.55	<.000 ***
extraction.solvent * purification.method	1	7.81	7.81	7.02	0.029 *
Residuals	8	8.9	1.11		

Table S2: **Method comparison.** Yield and stable hydrogen isotopic composition of sucrose octaacetate from bean leaves *(Vicia faba)* across different methods of extraction solvent (water or 80%-ethanol) and purification (solid phase extraction or liquid-liquid separation). The yield was assessed as the amount of sucrose octaacetate obtained after purification (measured on a GC-FID) per initial dry weight. The hydrogen isotopic composition was then measured on a GC-IRMS and is stated as δ^2H with associated standard deviation (SD) obtained from repeated measurements (n).

**Sample replicates**	**Extraction solvent**	**Purification method**	**Yield per initial dry weight (µg/mg)**	**Stable isotope values of non-exchangeable hydrogen**		
				**δ^2H (‰ VSMOW)**	**SD from replicate injections**	**n**
1	Water	Liquid-liquid separation	4.9	-162	0	2
2			7.3	-160	0	2
3			3.9	-167	1	2
		**Average ± SD:**	**5.4 ± 1.8**	**-163 ± 3**		
1	Water	Solid phase extraction	0.7	-157	3	2
2			0.7	-170	0	2
3			0.7	-162	3	2
		**Average ± SD:**	**0.7 ± 0**	**-163 ± 6**		
1	80%-Ethanol	Liquid-liquid separation	17.3	-163	0	2
2			15.9	-160	0	2
3			17.2	-166	0	2
		**Average ± SD:**	**16.8 ± 0.8**	**-163 ± 3**		
1	80%-Ethanol	Solid phase extraction	9.5	-170	0	2
2			9.4	-164	2	2
3			8.1	-165	0	2
		**Average ± SD:**	**9.0 ± 0.8**	**-166 ± 4**		
		**Total average ± SD:**	**8.0 ± 6.2**	**-164 ± 4**		

Figures

Figure S1: **Effect of pyridine purity on GC-IRMS chromatography and δ^2H values.** Whole *Vicia faba* leaf carbohydrate extracts were acetylated with acetic anhydride and pyridine. For the pyridine, either a high purity pyridine (≥ 99.5 %, panel A and yellow points in panel C) or a low purity pyridine (≥ 99.0 %, panel B and purple points in panel C) was used. Note the hump in the chromatography (B) at the end of the sequence when low purity pyridine is used, and the difference in the δ^2H values of sucrose octaacetate (C).

Figure S2: **Effect of SPE sorbent and pH on sucrose and glucose recoveries.** Three SPE sorbents (HR-X, C8ec, C18ec) were tested with stock sucrose octaacetate (A) and glucose pentaacetate (B) (200 µg each per sample). To simulate the solvent system, 5 mL each of acetic anhydride and pyridine were added, and samples were heated for 2 h at 50 °C. Then, samples were diluted and either acidified to pH 2 using HCl (purple bars) or not (pH 5, yellow bars). Recoveries were calculated as the percentage of the compound measured by GC-FID relative to its initial amount. Shown are the mean and standard deviation of n = 2-3 replicates.

Figure S3: **Effect of SPE sorbent and dilution volume on sucrose yields.** Three SPE sorbents (HR-X, C8ec, C18ec) were tested on acetylated carbohydrate extracts from bean leaf powder (extracted with a hot water extraction). Additionally, samples were diluted after derivatization with either 25 or 50 mL of nanopure water (blue or yellow bars, respectively). Yields were calculated as the amount of sucrose octaacetate measured by GC-FID relative to the initial plant dry weight. Shown are the mean and standard deviation of n = 2-3 replicates.

Figure S4: **GC-FID chromatogram of a solvent blank.** Acetic anhydride and pyridine (5 mL each) were heated for 2 h at 50 °C, diluted with 50 mL ultrapure water, applied to SPE columns, and eluted with 20 mL acetone after the solution was eluted to the top of the stationary phase. A chromatogram of pure sugar acetates is added in grey as reference.

Figure S5: **Assessment of sample composition.** GC-MS chromatogram of a carbohydrate extract from bean leaf material (*Vicia faba*) extracted with 80%-ethanol, and after acetylation subsequently SPE-purified (A). The main peaks are numbered and the region of monosaccharides and sucrose highlighted with grey boxes and enlarged in panels B and C. The peaks were identified as following substances: 1) mannitol-acetate, 2) siloxane,

3) 3,7,11,15-Tetramethyl-2-hexadecen-1-ol, 4) methyl-tetraacetyl-α-D-mannopyranoside, 5+7) D-glucose-pentaacetate, 6) D-Galactofuranose-pentaacetate, 8) myo-inositol-hexaacetate, 9) tetracosanol, 10) cellobiose-octaacetate, 11+13) sucrose-octaacetate, 12) acetylated glucopyranoside.

Figure S6: **Example chromatograms of acetylated plant carbohydrate extracts purified with the new SPE method.** Shown are chromatograms of soluble sugar extracts and starch extracts. These were produced from leaves of radish *(Raphanus sativus)* and sunflower *(Helianthus annuus)*, leaves and roots of tobacco *(Nicotiana sylvestris),* as well as leaves and wood of several tree species *(Betula, Carpinus, Fagus)*. Species, plant organ, and sample type ("sucrose" for soluble sugar extracts, or "starch" for starch extracts) are indicated in the header of each chromatogram.

Figure S7: **Example chromatograms of acetylated plant carbohydrate extracts with high monosaccharide contents.** Shown are chromatograms of soluble sugar extracts from wild-type and *pgm*-mutant *Nicotiana sylvestris* leaves which contained a high ratio of monosaccharides to sucrose, complicating chromatography.

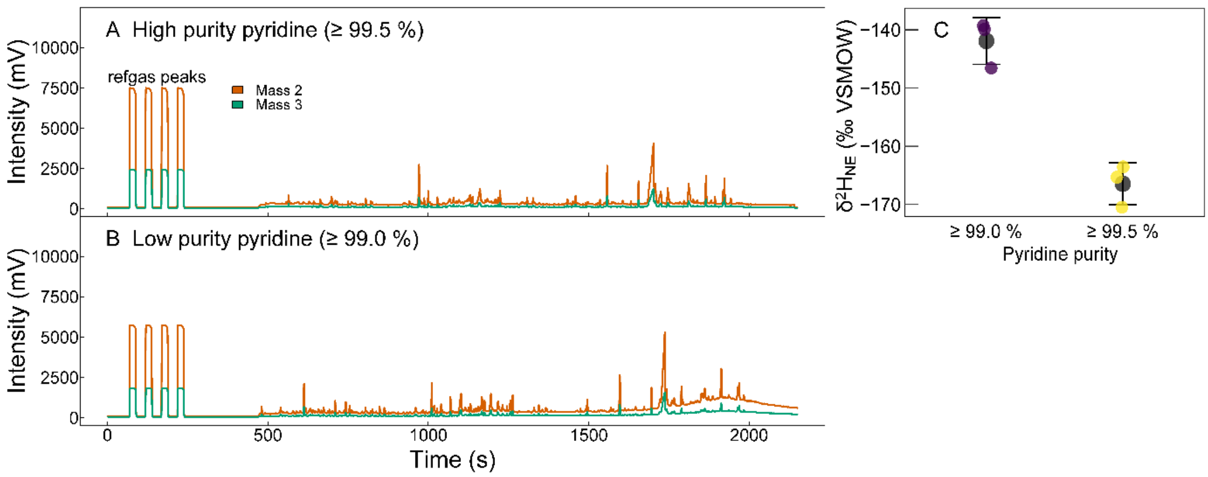

Figure S1

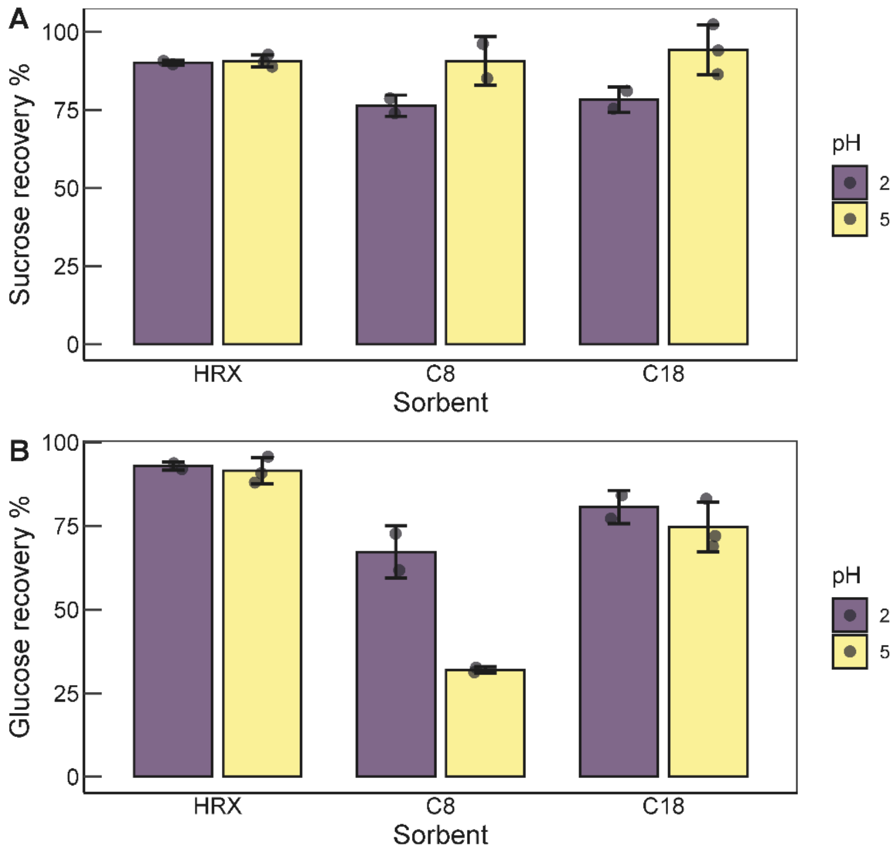

Figure S2

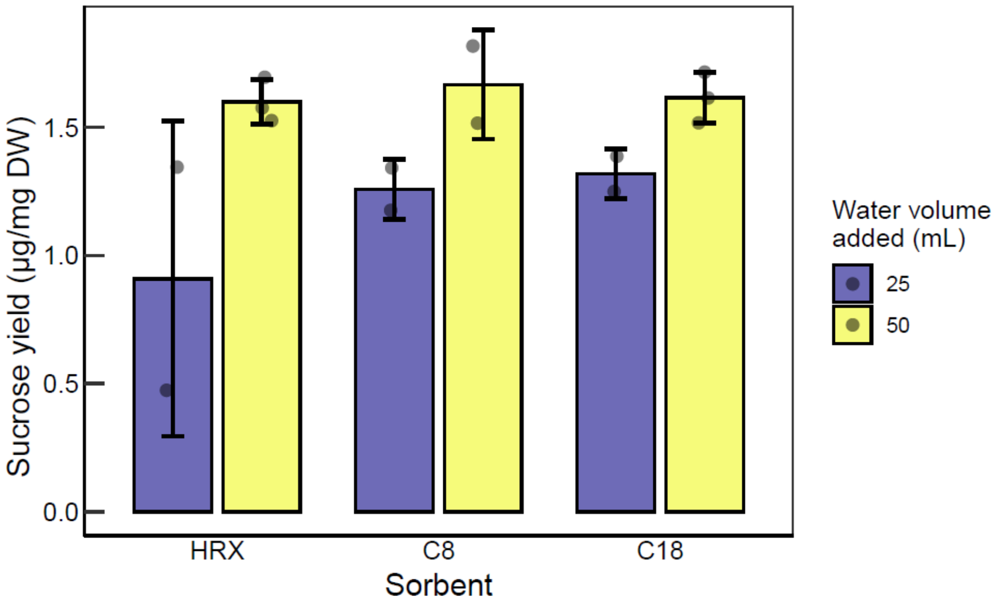

Figure S3

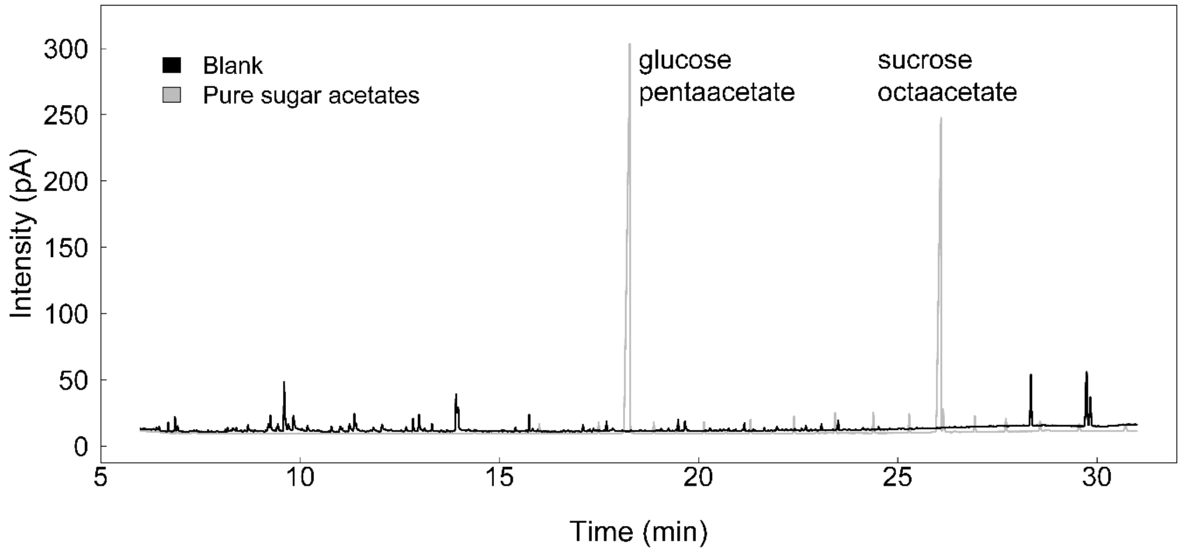

Figure S4

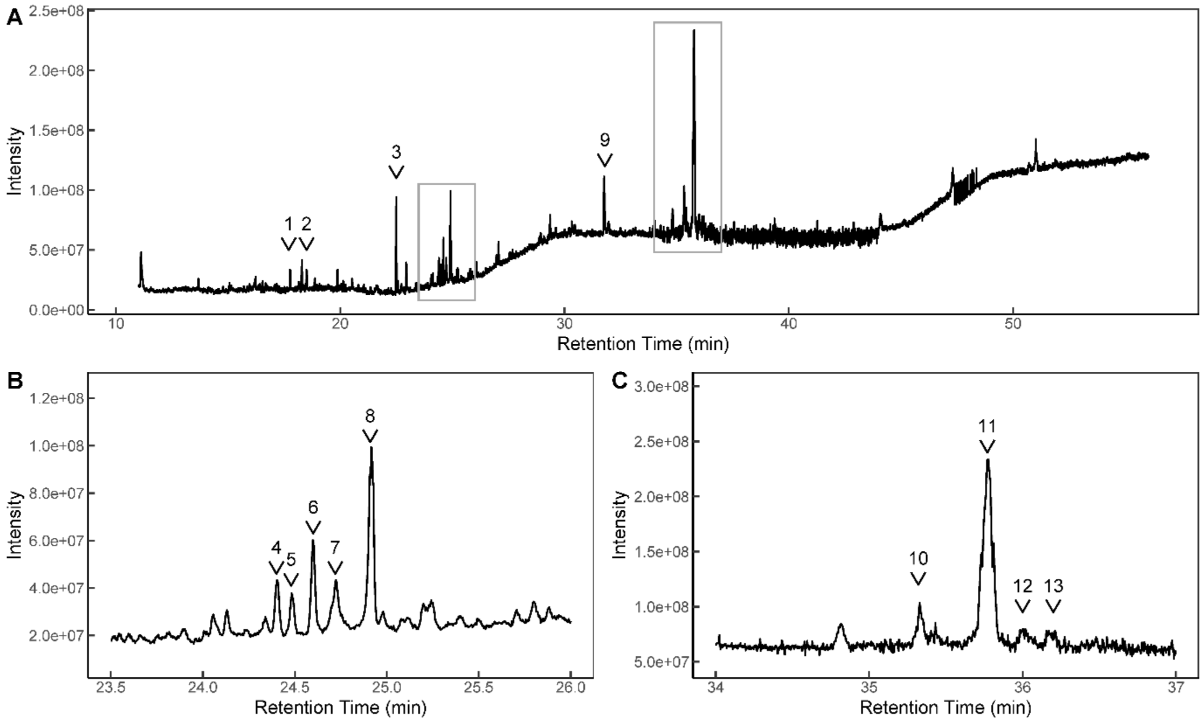
A
B
C
Intensity
Retention Time (min)
2.5e+08
2.0e+08
1.5e+08
1.0e+08
5.0e+07
0.0e+00
10
20
30
40
50
1
2
3
9
1.2e+08
1.0e+08
8.0e+07
6.0e+07
4.0e+07
2.0e+07
23.5
24.0
24.5
25.0
25.5
26.0
4
5
6
7
8
3.0e+08
2.5e+08
2.0e+08
1.5e+08
1.0e+08
5.0e+07
34
35
36
37
10
11
12
13

Figure S5

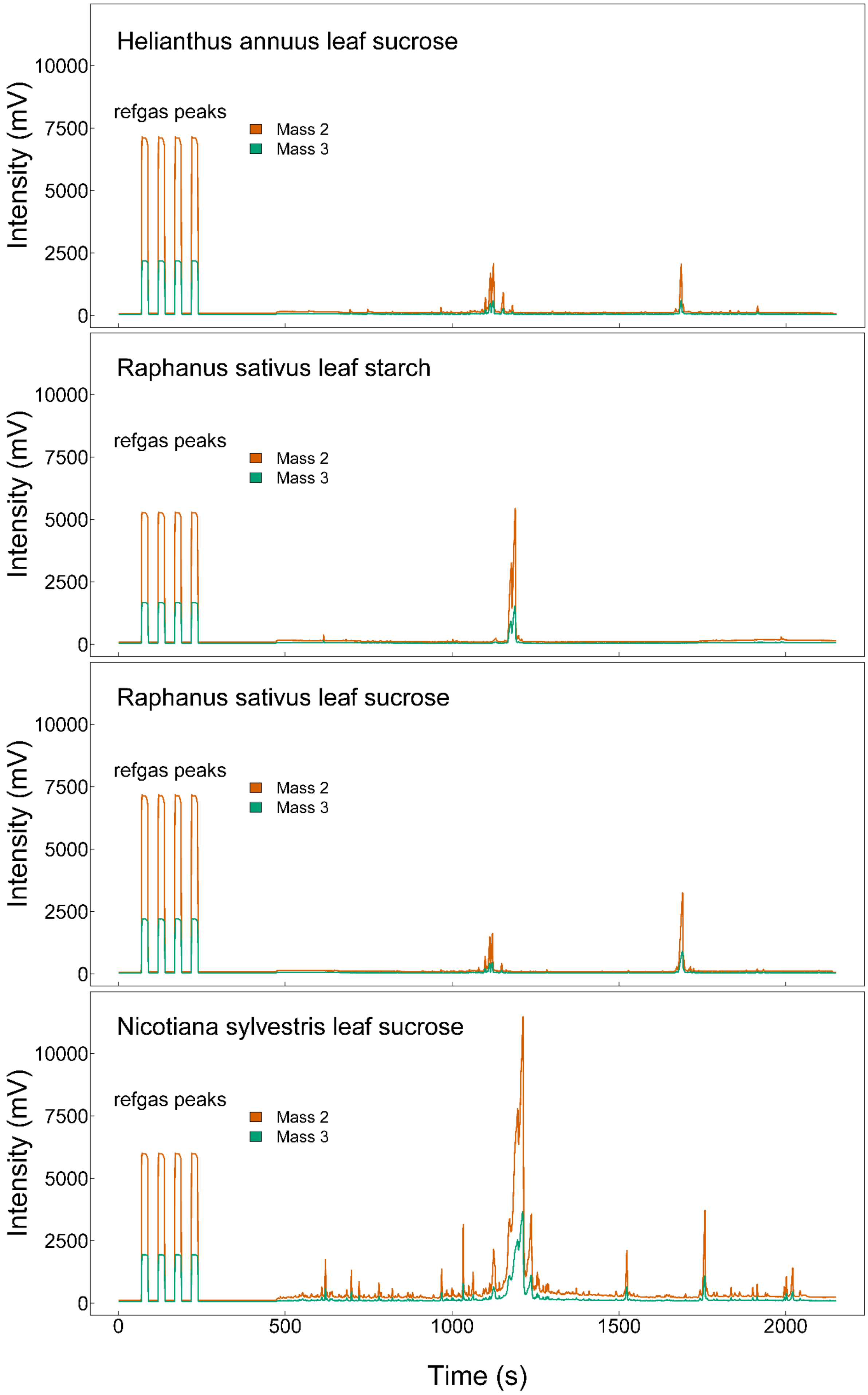
Helianthus annuus leaf sucrose
Raphanus sativus leaf starch
Raphanus sativus leaf sucrose
Nicotiana sylvestris leaf sucrose
refgas peaks
Mass 2
Mass 3
Intensity (mV)
0
2500
5000
7500
10000
Time (s)
0
500
1000
1500
2000

Figure S6

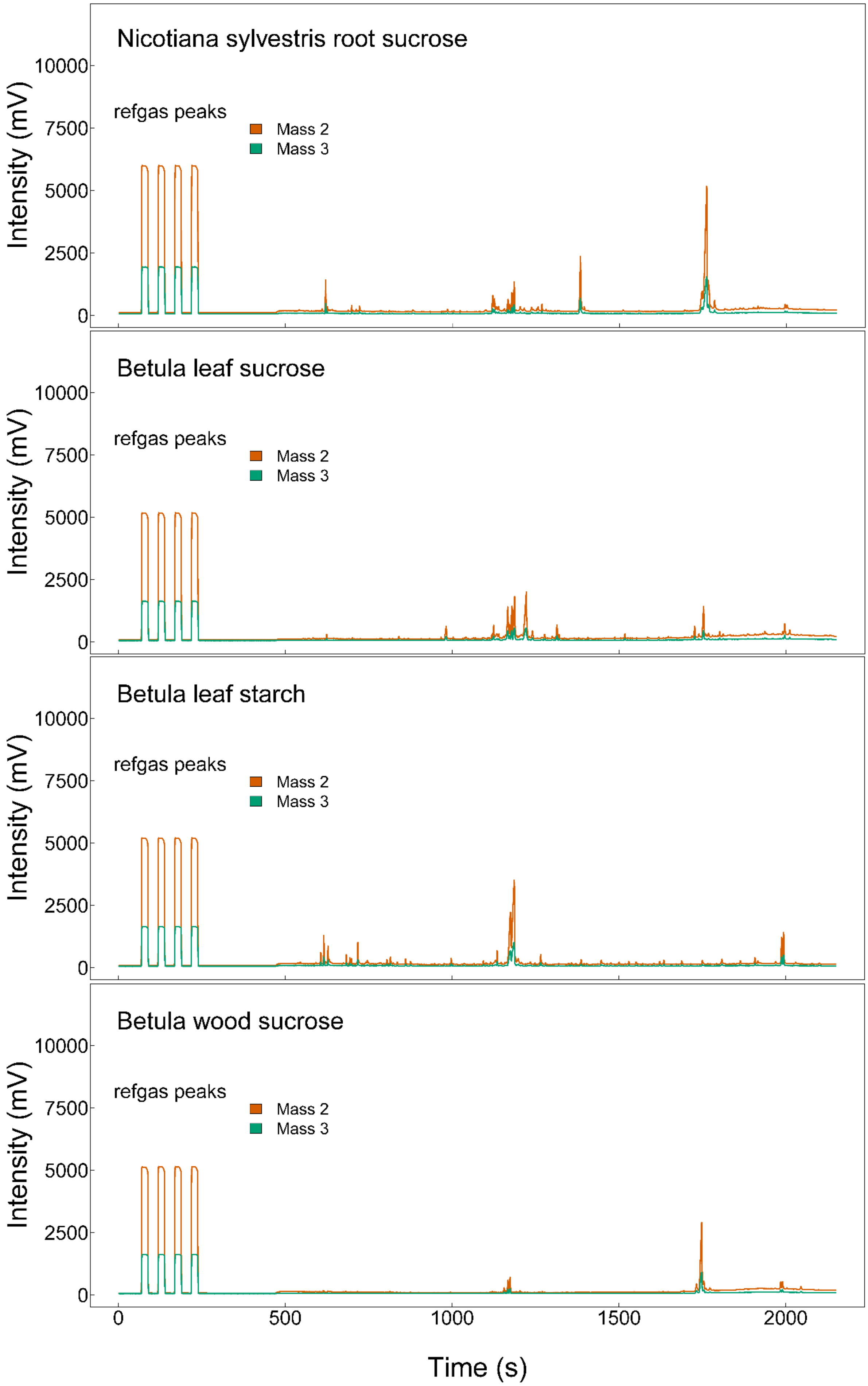

Figure S6 (continued)

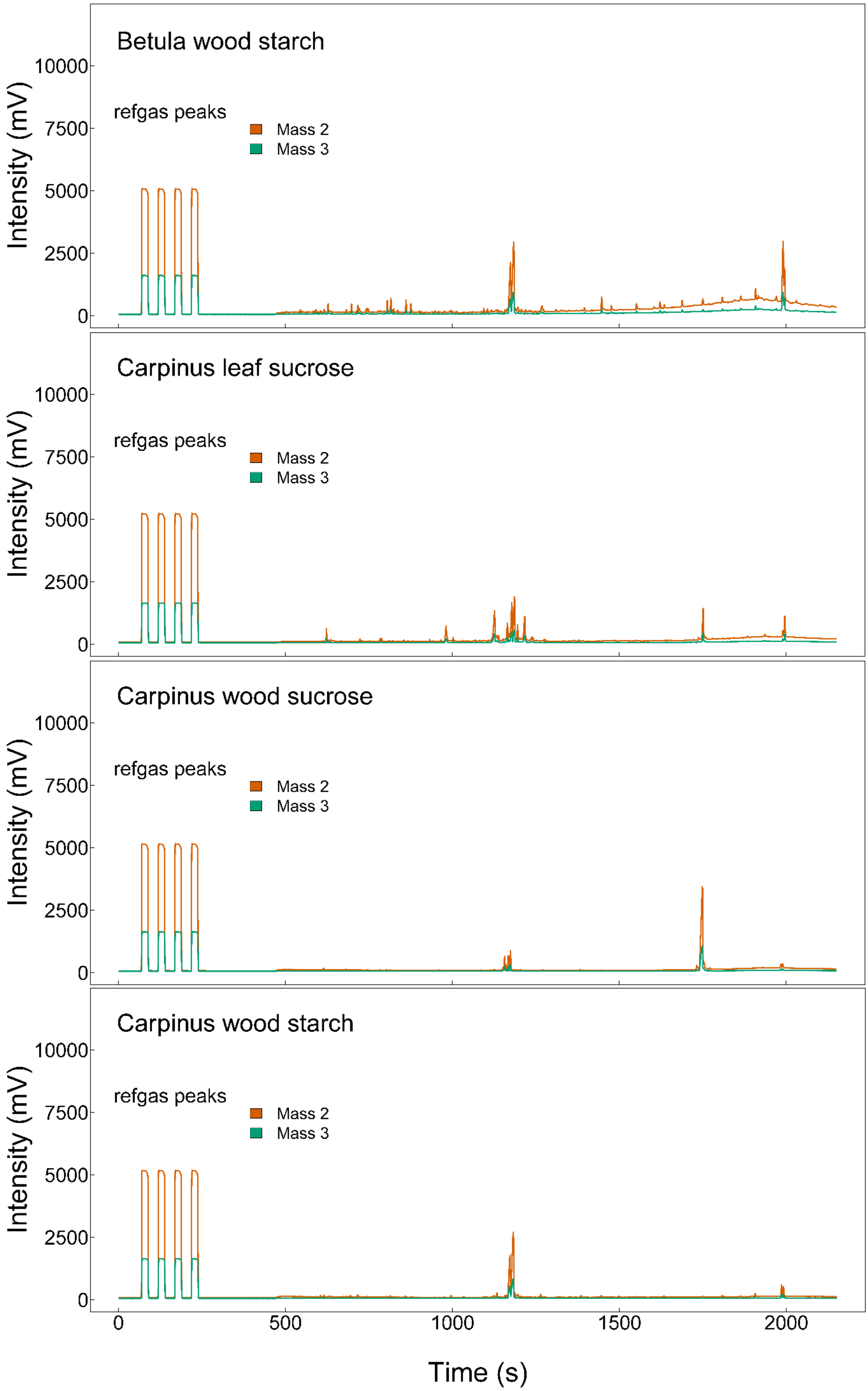

Figure S6 (continued)

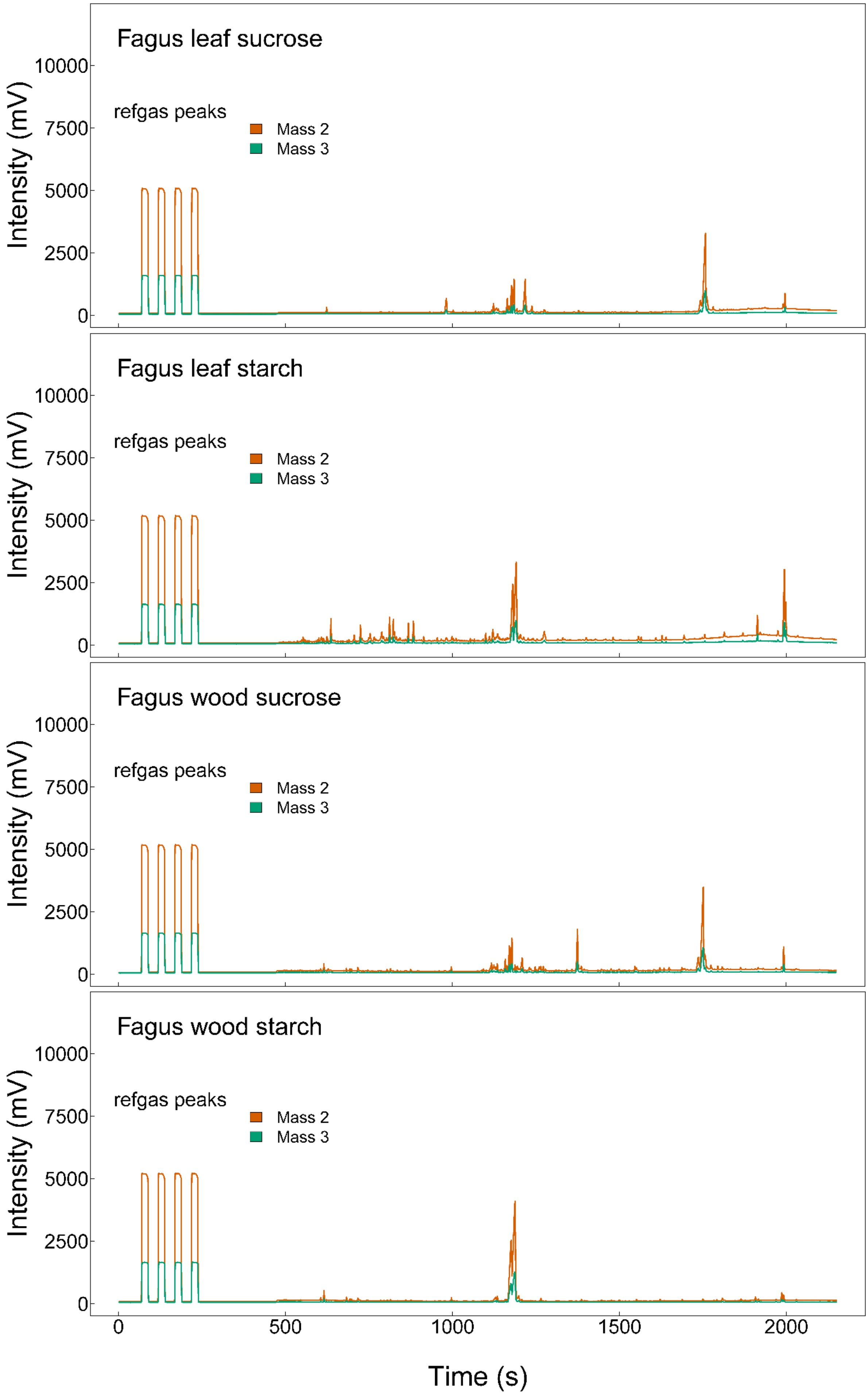

Figure S6 (continued)

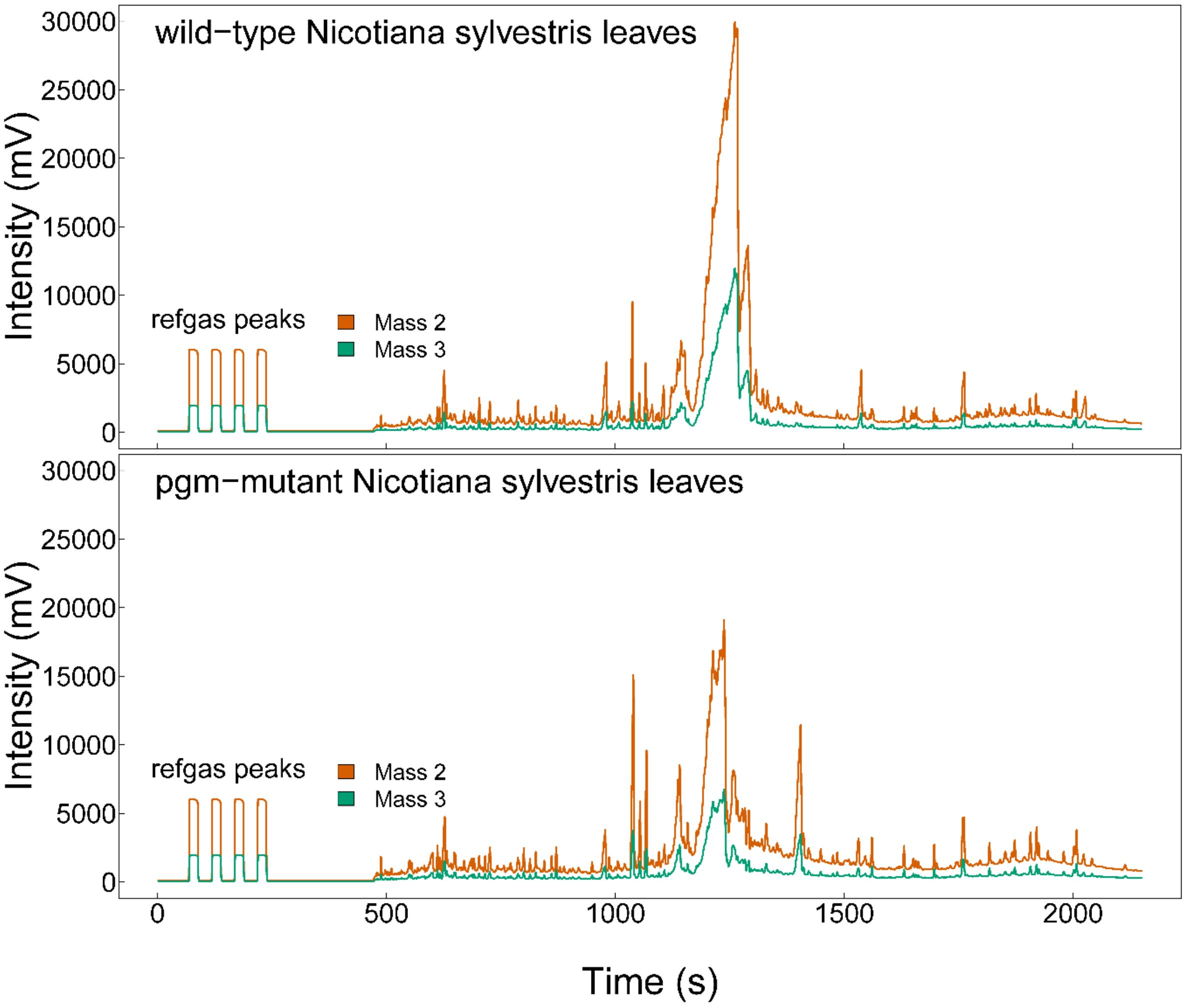
wild−type Nicotiana sylvestris leaves
pgm−mutant Nicotiana sylvestris leaves
refgas peaks
Mass 2
Mass 3
Intensity (mV)
30000
25000
20000
15000
10000
5000
0
500
1000
1500
2000
Time (s)

Figure S7
